# Neural mechanisms of spatial navigation in ASD and TD children: insights from EEG microstate and functional connectivity analysis

**DOI:** 10.3389/fpsyt.2025.1552233

**Published:** 2025-04-04

**Authors:** Yao Wang, Jianing Wang, Chong Lu

**Affiliations:** ^1^ Cognitive Science and Allied Health School, Beijing Language and Culture University, Beijing, China; ^2^ Institute of Life and Health Sciences, Beijing Language and Culture University, Beijing, China; ^3^ Key Laboratory of Language and Cognitive Science (Ministry of Education), Beijing Language and Culture University, Beijing, China; ^4^ Department of Rehabilitation Medicine, Beijing Jianjia Rehabilitation Hospital, Beijing, China; ^5^ College of International Education, Minzu University of China, Beijing, China

**Keywords:** autism spectrum disorder, microstates, functional connectivity, cognition, navigation

## Abstract

**Introduction:**

Autism Spectrum Disorder (ASD) is associated with atypical neural dynamics, affecting spatial navigation and information integration. EEG microstates and functional connectivity (FC) are useful tools for investigating these differences. This study examines alterations in EEG microstates and theta-band FC during map-reading tasks in children with ASD (n = 12) compared to typically developing (TD) peers (n = 12), aiming to uncover neural mechanisms underlying spatial processing deficits in ASD.

**Methods:**

EEG data were collected from children with ASD (n = 12) and TD controls (n = 12) aged 6-10 years during a map-reading task. Microstate analysis quantified the temporal dynamics of four canonical microstates (A, B, C, and D). Theta-band (4-8 Hz) FC was analyzed to assess interregional neural communication during the task. Statistical tests identified group differences in microstate metrics and FC patterns.

**Results:**

Children with ASD showed significant differences in EEG microstate dynamics compared to TD controls. The ASD group showed reduced occurrence, but longer duration and greater coverage in microstate A, indicating abnormal temporal and spatial brain activity. For microstate B, the ASD group displayed shorter durations and lower coverage, suggesting impairments in cognitive control. In microstate C, the ASD group exhibited reduced duration, coverage, and steady-state distribution, pointing to disruptions in spatial attention. Conversely, microstate D showed increased occurrence and greater coverage in the ASD group, reflecting atypical spatial attention allocation. Theta-band FC analysis revealed significantly reduced connectivity in key brain networks involved in spatial navigation, particularly between fronto-parietal and occipito-temporal regions. This suggests disrupted integration of spatial and cognitive processes in children with ASD.

**Discussion:**

The alterations in EEG microstate patterns and theta-band FC highlight differences in the neural mechanisms underlying spatial navigation and cognitive integration in ASD. These findings suggest that microstate and FC analyses could serve as biomarkers for understanding visual spatial navigation in ASD, related to perceptual abnormalities. This research provides a basis for individualized rehabilitation plans for children with ASD, using qEEG biomarkers to guide neuromodulation techniques, such as transcranial direct current stimulation (tDCS). Future studies should investigate longitudinal changes and intervention effects on these neural dynamics.

## Introduction

The brain’s instantaneous global functional state can be reflected in its electric field configuration. Transient changes in this configuration, termed EEG microstates, represent descriptions of the brain’s sequential electric field phenomena. Microstates are short-lived, quasi-stable topographical segments lasting approximately 60–120 milliseconds. These segments undergo rapid transitions between distinct metastable states (1). The dynamic changes in microstates are crucial mechanisms for neural information processing and are strongly associated with cognitive functions as well as various neurological and psychiatric disorders (2, 3). The temporal dynamics of brain processes described by microstates align with several theoretical perspectives. These theories propose that consciousness and cognition are temporally discontinuous, a notion supported by numerous electrophysiological and imaging studies (4). The discrete changes in microstates may be driven by distinct neural mechanisms. Four primary EEG microstates, labeled A through D, have been identified. Each microstate is associated with specific neural activity patterns, reflecting the distinct brain states during the processing of various types of information (5). This study aims to investigate differences in EEG microstate characteristics during map processing tasks between children with Autism Spectrum Disorder (ASD) and children with typically developing (TD). Specifically, we compared the coverage and duration of microstates A, B, C, and D between the two groups. We hypothesize that during map processing tasks, ASD children will exhibit distinct microstate activity patterns compared to TD children. These differences may reflect unique neural mechanisms underlying the visual spatial and navigation processing in children with ASD. Understanding the neural correlates of map processing in ASD children holds significant implications for assessing their cognitive processing capacities and spatial navigation abilities. Furthermore, this study may give a new perspective for detecting the reason that people with ASD are overly sensitive to environment and space changes We proposed that the special mechanisms of spatial cognition reflected by the map processing tasks the current study applied may cause the stereotyped behaviors of ASD related with the physical and social environments changes. And help to reveal the cognitive mechanisms underlying the integration of perceptual and conceptual information during map processing tasks with ASD. In summary, this research aims to provide valuable insights into the neural basis of map processing in ASD children and its potential impact on spatial information processing abilities. <The findings could inform the development of targeted interventions to enhance map cognition and navigation skills in the ASD population and could further give some implications of new methods for disordered perceptual and behavioral rehabilitations and estimation with ASD>.

Microstates are increasingly recognized as fundamental components of cognition, serving as potential neural correlates of human thought processes (2, 6). These transient, quasi-stable brain electric field topographies, lasting approximately 60–120 ms, have been described as the “atoms of thought” and are modulated by the content of an individual’s cognitive states (6, 7). As such, microstates are considered the basic building blocks of human cognition and consciousness, with their characteristics reflecting the dynamic interplay of mental states. Lehmann et al. (6) first observed that microstates can be influenced by the content of spontaneous thoughts during resting-state EEG recordings. Participants were asked to report their thoughts after auditory cues, and the results indicated significant differences in microstate topographies corresponding to distinct categories of thought, demonstrating that microstates are indeed shaped by mental states. Furthermore, the temporal dynamics of microstates are consistent with the subsecond time scales associated with the large-scale synchronization of neural networks (8). Extensive studies have also linked microstates to various neuropsychiatric and neurological disorders that lead to cognitive impairments (9, 10). Research has highlighted the relationship between microstates and cognitive functions, suggesting that they serve as an important index for cognitive processing. For example, Britz et al. (11) combined EEG with functional magnetic resonance imaging (fMRI) to explore the neural correlates of four primary microstates (A, B, C, and D). These microstates correspond to specific functional brain networks identified in resting-state fMRI studies: Microstate A is linked to the auditory network, Microstate B to the visual network, Microstate C to cognitive control and partially to the default mode network (DMN), and Microstate D to the dorsal attention network (12). Microstates B and C, in particular, are crucial for visual processing and cognitive control, respectively, and have been shown to be highly sensitive to changes in external sensory input. Seitzman et al. (5) found that increased visual input significantly alters the temporal characteristics of Microstate B, manifesting as an increase in its coverage and occurrence, supporting the hypothesis that Microstate B is closely related to visual processing. Moreover, Microstate C has been associated with self-referential processing, a characteristic of the anterior default mode network, and plays a key role in the cognitive control. Britz et al. (11) linked Microstate C to the salience network, including regions such as the anterior cingulate cortex and insula, which are crucial for task execution and attentional control (13). Additionally, research has shown that microstates can offer valuable insights into emotional processing. Microstate C exhibits increased coverage and duration during the neutral emotional states, while Microstate B is predominantly linked to the negative emotions (14). This suggests that microstates are modulated not only by cognitive processes but also by emotional states, highlighting their role as a comprehensive brain dynamic representing of human cognition and emotion. Based on these insights, the current study aims to explore the differences in microstate dynamics between two groups with distinct cognitive profiles: TD children and children with ASD. Previous research suggests that individuals with ASD may display atypical neural processing during cognitive tasks, as evidenced by altered microstate patterns compared with typically development individuals. Specifically, Microstates B and C, which are related to visual processing and cognitive control, are expected to play critical roles in spatial navigation and information integration. In this context, the current study investigates how these microstates are modulated during the map-based navigation tasks, examining differences in coverage, occurrence, and duration of Microstates A, B, C, and D between TD and ASD groups.

In addition to examining EEG microstates, functional connectivity (FC) analysis provides the important perspectives on the interactions between brain regions during cognitive tasks. FC reflects the temporal correlations between brain activity across distant cortex areas, offering insights into the integration of information across large-scale neural networks (15). Theta waves (4–8 Hz) are particularly relevant in this context, as they are associated with cognitive functions such as memory, attention, and spatial navigation (16, 17). The hippocampus, a region integral to spatial memory, exhibits strong theta oscillations during tasks involving spatial navigation, such as map processing (18). Previous studies have shown that theta waves support the integration of information between brain regions involved in spatial cognitive processing, such as the hippocampus, parietal cortex, and prefrontal cortex (19, 20). In individuals with ASD, there is evidence of altered theta activities, particularly in regions involved in set-shifting and working memory for spatial information (21, 22). These altered connectivity patterns may underlie difficulties in cognitive tasks, such as spatial navigation, that require the integration of lots of information. Research suggests that reduced theta connectivity in regions such as the hippocampus and visual cortex may impair the ability to integrate spatial and visual information (23), contributing to the challenges in map processing observed in children with ASD. Therefore, theta FC analysis is a critical component of understanding the neural mechanisms underlying the spatial processing deficits in ASD. The focus on the theta oscillation functional connectivity in this study is grounded in its role in coordinating brain networks during tasks requiring spatial memory and navigation. We hypothesize that children with ASD will exhibit disrupted or reduced theta-band connectivity between key brain regions during the map processing tasks, such as the hippocampus, visual cortical areas, and regions associated with the cognitive control compared with the normal groups (23). This connectivity analysis of theta oscillation will be the fine complements for the microstate dynamics examination, providing a comprehensive view of how brain networks interact during map-based tasks and how these dynamics may differ between children with ASD and TD. By investigating theta connectivity alongside microstates, we aim to further elucidate the functional integration deficits observed in ASD and their impact on spatial information processing. Microstates share a high degree of similarity with several cognitive theories of consciousness. The concept of perceptual frames was introduced by Efron to describe the dynamic changes in brain activity over time, which reflect the brain’s information processing. Perceptual frames suggest that the temporal dynamic of the brain activities involves a sequence of processes, including the segmentation, the encoding, the structuring, and the decoding, that transform and modulate the input information.

In perceiving and understanding external information, the brain operates according to this temporal–dynamic framework, where cognitive processes are organized and adjusted across different stages to handle and reassemble the afferent information. This description emphasizes the importance of time sequence during cognitive process and reveals the details of how the brain organizes and interprets the afferent information over time, leading to external perception and understanding. Microstates can be divided into different segments of stable topographies, with each segment lasting a certain period of time, while the transitions between stable states occur rapidly. This aligns well with the temporal dynamics described in the perceptual frame theory (1, 4). Dehaene and Changeux’s (24) neural workspace model provides a theoretical framework for understanding brain consciousness. This model divides the neural workspace into different regions, each representing distinct states of consciousness. When important information is processed in some local brain areas, it is transmitted to the global workspace and widely distributed across it, involving competition and selection of conscious content. Microstates, as the “atoms of thought,” represent the basic components of consciousness and exhibit random variations (25–27). By observing changes in microstate activity, we can attempt to understand the differences and transitions between various states of consciousness. Studies related to navigation tasks have shown that specific neurophysiological signals are influenced by cognitive control. Research indicates that theta oscillations play the important role in combining the separated events over long time scales in navigation and especially in memory retrieval and other information processing (28). Watrous et al. (29) used EEG and brain imaging techniques to investigate neural activity during navigation tasks, indicating that enhanced network connectivity in specific frequency band was closely related to accurate spatial memory retrieval. Jacobs et al. (30) found that gamma oscillations in the right neocortex play a unique role in human spatial navigation. Kaplan et al. (31) demonstrated that theta-gamma phase coupling is crucial for human spatial memory, with oscillations in these two frequency bands being closely associated with the spatio-temporal characteristics of navigation behavior. Existing research has demonstrated that subjects with more prior knowledge related with navigation showed higher accuracy in the process of route planning with stronger functional connectivity between the frontal and temporal lobes in EEG signals compared to the subjects without any navigation related knowledge during the map navigation experiment (32). EEG activity provides specific indicators for processing and executing navigation information. Therefore, based on these findings, we hypothesize that children with TD and children with ASD may exhibit differences in EEG function when processing map information. As an instantaneous reflection of global brain function, microstates offer a unique perspective on the brain’s information processing mechanisms. Specifically, microstates B and C are closely associated with visual and autonomous processing, respectively. The current study aims to investigate the specific characters represented by EEG functional states of children with ASD compared with typical development controls during map information processing tasks. <Based on previous studies, there are significant differences in brain functional connectivity during map information processing (32), and we hypothesize that similar differences will also be observed in ASD participants when processing map information. The current study intend to focus on exploring EEG microstates during visual map information processing, particularly the cognitive functions of microstates A, B, C, and D in visual and autonomous processing. These microstates are believed to reflect the transient functional configuration of large-scale neural networks, providing important insights into underlying cognitive processes.

## Method

### Participants

A total of 24 children participated in this study, aged between 6 and 10 years (M = 7.2, SD = 1.61). Twelve children with high-functioning autism spectrum disorder (ASD) were clinically recruited from pediatric developmental or psychiatric departments, with diagnoses confirmed by experienced clinicians. All ASD participants met the following inclusion criteria: no comorbid neurological disorders (e.g., epilepsy), intellectual disability, visual impairment, or hearing impairment, and had an IQ greater than 70. These high-functioning children with ASD were also recommended by experienced speech-language pathologists and demonstrated good cognitive executive functioning in their regular interventions.

Additionally, 12 typically developing (TD) children, matched with the ASD group by age and gender, were recruited for comparison. All TD participants had normal vision and hearing, no history of psychiatric disorders, and an IQ greater than 70. Informed consent was obtained from the legal guardians of all participants, who also received appropriate compensation for their participation. This study was approved by the Ethics Committee of Beijing Language and Culture University (Approval Number: 2024BYLL25).

### Task

In this experiment, participants were required to complete a route-planning task followed by passive observation of a map. At the beginning of the task, a starting point (A), an endpoint (B), and a pre-planned route were marked on the map. Participants were instructed to trace the specified route from point A to point B using a pen, navigating based on the provided transportation network. The task emphasized selecting the quickest and shortest path, requiring participants to identify and trace the most efficient route. Due to individual differences in the time taken to complete the task, each trial typically lasted between 30 seconds and 1 minute. After completing each trial, participants would begin another task of finding the optimal path again to meet the required time for data collection. This was done until the total EEG data collection time reached a minimum of three minutes. The entire procedure was uninterrupted, ensuring that the EEG data duration was consistent across all participants.

### EEG recording

Participants were seated comfortably in a chair and presented with a map of Beijing measuring 1.1 meters by 0.8 meters. Electroencephalogram (EEG) data were collected during a three-minute session using the Electrical Geodesics Inc. (EGI) EEG system. The system included a geodesic sensor net, a Net Amps amplifier, and Net Station software for data acquisition and analysis. A 65-channel EEG cap was utilized, with a data sampling rate of 1000 Hz and analog bandpass filtering set between 0.1 and 200 Hz. All electrode impedances were maintained below 50 KΩ before initiating data collection. Real-time EEG signals were monitored using the Net Station acquisition software provided by EGI.

### Data analysis

To investigate the FC between brain regions, we performed a correlation-based analysis of the theta rhythm (4–8 Hz) in the EEG data. EEG signals were preprocessed to isolate the theta rhythm using a bandpass filter. Specifically, the data were filtered between 4 and 8 Hz using a finite impulse response (FIR) filter, a standard method for isolating theta activity. This frequency band is commonly associated with cognitive functions such as attention and memory. The filtered data were then averaged across trials to minimize variability from different experimental conditions and focus on the general neural response. For each subject, we calculated the correlation coefficient matrix between the EEG signals from all pairs of channels. This matrix captures the degree to which the activity of one brain region (represented by a channel) correlates with that of another, providing insights into the functional relationships between brain regions. Specifically, the correlation matrix was computed by calculating Pearson’s correlation coefficients for the averaged theta rhythm signals from each channel. To focus on meaningful connections, we retained only those pairs of channels with a correlation coefficient greater than 0.7, reflecting a moderate to strong functional relationship between regions. This threshold was chosen based on prior research suggesting that correlations above 0.7 are typically indicative of significant functional interactions in EEG studies (33). Additionally, connections with a correlation coefficient greater than 0.9 were highlighted to emphasize the strongest relationships between brain regions. To calculate the global functional connectivity mean (FC_mean) for each subject, we used the following formula:


$$FC_{\text{mean}}=\frac{1}{N}\sum r_{ij}$$

Where:

- *r_{ij}_* represents the correlation coefficient for each pair of channels in the correlation matrix.
- *N* is the number of off-diagonal elements in the matrix (i.e., the number of unique channel pairs). This excludes the diagonal elements, which represent the correlation of a channel with itself and are not meaningful for this analysis. The global functional connectivity mean provides a single value that summarizes the overall level of connectivity between brain regions for each subject. This measure reflects the average strength of functional interactions across the entire network of brain regions, capturing both moderate and strong correlations. To statistically compare functional connectivity between the ASD and TD groups, we performed a standard t-test or non-parametric tests (such as the Wilcoxon rank-sum test), depending on the data distribution. The t-test was applied to the global functional connectivity means for each group to identify any significant differences in overall functional connectivity between the groups. This analysis method is particularly useful for exploring the complex interactions between brain regions in a frequency-specific manner. By focusing on the theta rhythm, which is known for its role in cognitive processing, particularly in attention and memory tasks, we aim to investigate how neural networks are organized and how these patterns may differ in cognitive states, such as those observed in ASD.

EEG data were preprocessed in MATLAB using the EEGLAB toolbox. The preprocessing steps involve artifact correction (using Automagic), band-pass filtering in the 1-30 Hz range (via pop_filtnew.m), and, if needed, epoch extraction for specific brain regions (using pop_epoch.m). Data selection and aggregation across subjects are done by concatenating EEG maps at GFP(Global Field Power) peaks, using the pop_micro_selectdata.m function. Key parameters include setting the data type to “Continuous”, applying average referencing and normalization, defining a minimum peak distance of 10 ms, a peak count of 1000, a GFP threshold of 1, and using datasets indexed from 1 to 4. During the microstate segmentation step, EEG maps are segmented at GFP peaks into microstates using the pop_micro_segment.m function. The modified k-means algorithm is applied, with the number of microstates set between 2 and 4, 50 repetitions, and optimization enabled. The topographies and fit measures of microstate prototypes are reviewed and the optimal number of microstates is selected using MicroPlotTopo.m and pop_micro_selectNmicro.m. Microstate prototypes are then imported into the datasets for back-fitting using pop_micro_import_proto.m. Finally, microstates are back-fitted and temporally smoothed using the pop_micro_fit.m and pop_micro_smooth.m functions, with smoothing set to “reject segments” and polarity set to 0. After all steps, microstate statistics are calculated with pop_micro_stats.m, and the results are exported for statistical analysis in SPSS. We primarily used the K-means algorithm, as referenced in studies (34, 35). For detailed procedures and programming code, please refer to the “Microstate EEGLAB Toolbox: An Introductory Guide” (35).

### Data statistics

The primary statistical analysis focused on the microstate data for A, B, C, and D. A repeated measures analysis of variance (ANOVA) was conducted, with the steady-state distribution, duration, occurrence, and coverage of microstates A, B, C, and D as the dependent variables. The within-subject factor was “Microstate Type (A/B/C/D),” while the between-subject factor was “Group (ASD/TD).” Effect sizes were calculated using partial eta-squared (η²) to assess the magnitude of the effects. All statistical analyses were performed using SPSS 26.0 software.

## Result

### Result of FC

**Figure 1:**
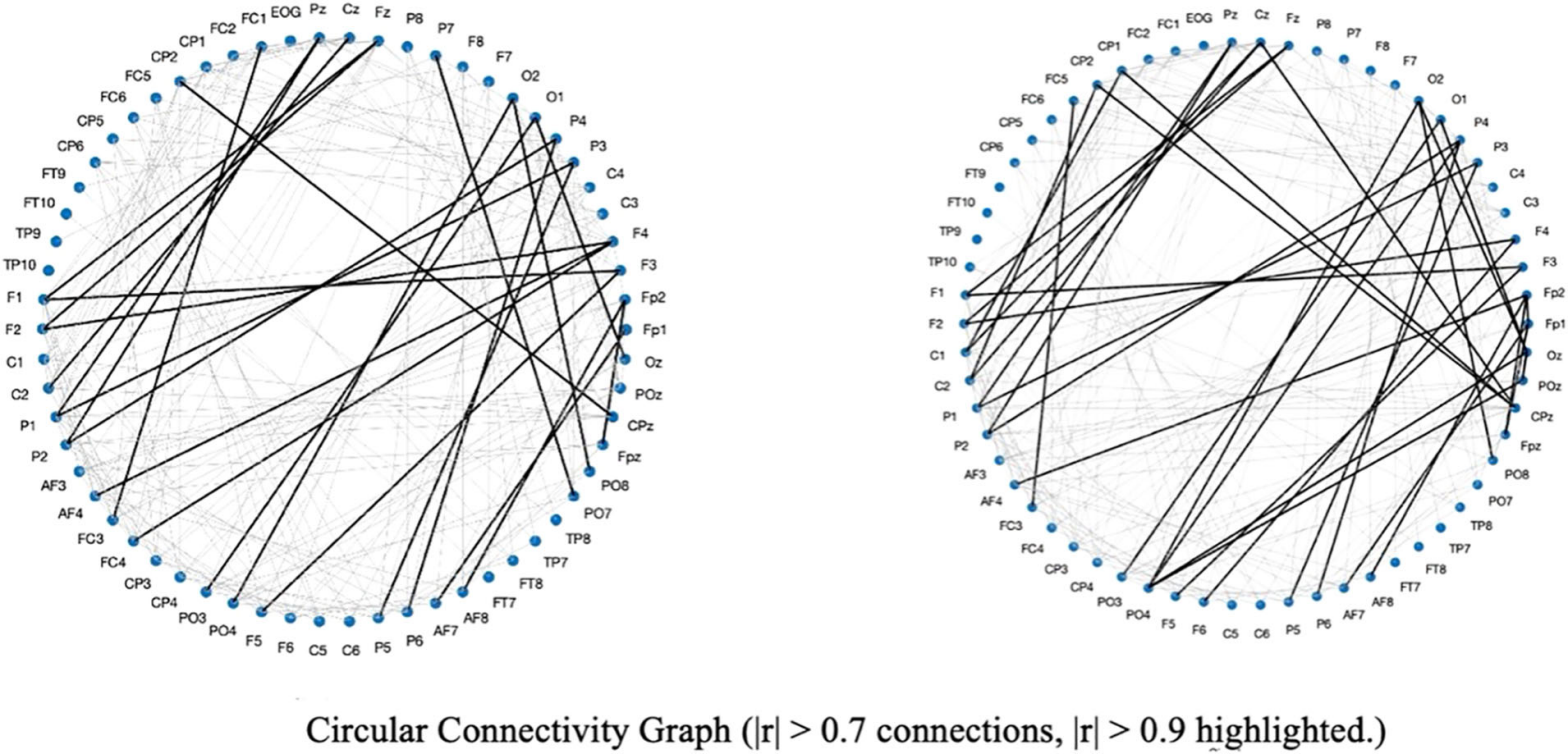
Circular connectivity graph illustrating functional connectivity patterns in the TD (left) and ASD (right) groups. Connections with |r| > 0.7 are shown, with stronger connections (|r| > 0.9)highlighted using thicker lines.

**Figure 2:**
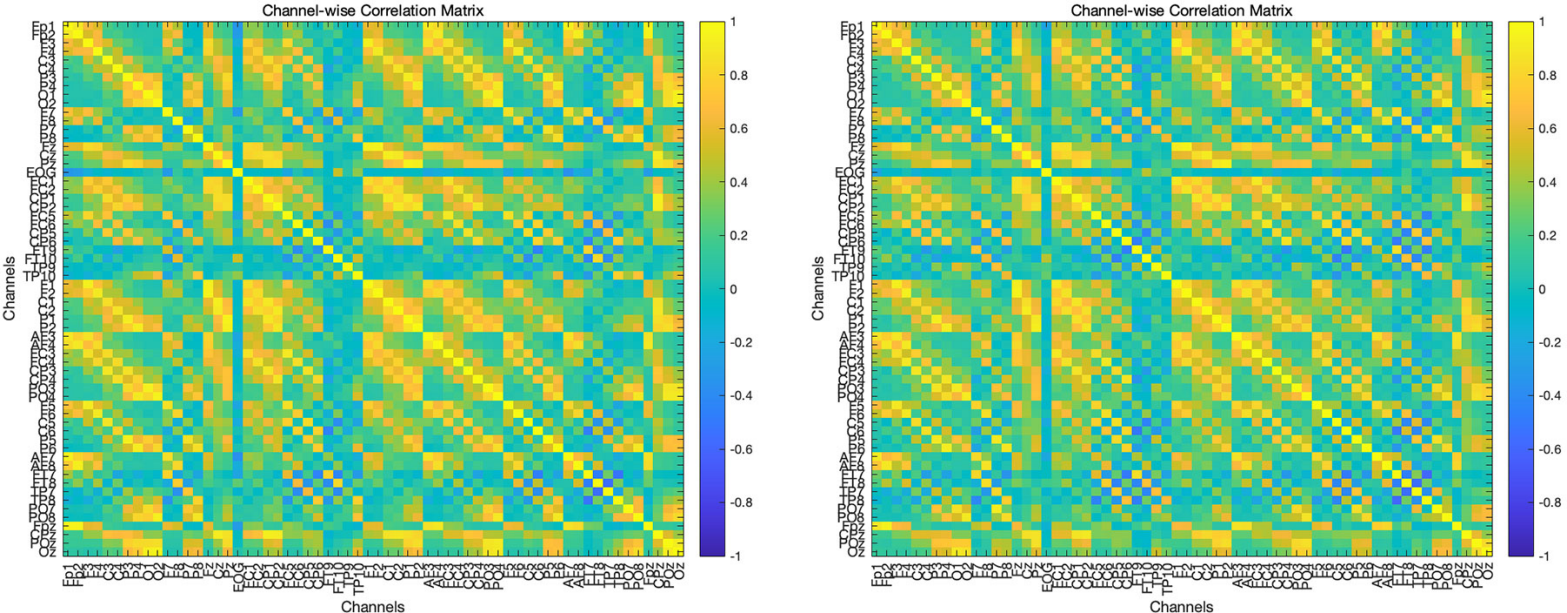
A correlation matrix illustrating functional connectivity patterns in the TD (left) and ASD (right) groups.

The t-test for global functional connectivity mean revealed a significant difference, with a t-statistic of *t* = -5.47 and *p* = 0.000051. The functional connectivity in children with ASD was significantly lower compared to the TD children. The results were visualized in two ways: a correlation matrix and a circular connectivity graph. The matrix provides a clear, numerical representation of the relationships between channels, where each cell indicates the strength of correlation between two channels. For the circular connectivity graph, a polar coordinate system was used to represent the channels as nodes placed on a circle, with connections between nodes drawn as lines. These connections were color-coded and sized according to their correlation values. Specifically, connections with a correlation coefficient greater than 0.9 were highlighted with thicker black lines, while connections with a correlation coefficient greater than 0.7 but less than 0.9 were drawn with thinner light gray lines, as shown in **Figures 1**, **2**.

The FC analysis revealed significant differences between the autism spectrum disorder (ASD) group and the typically developing (TD) group. Specifically, individuals with ASD exhibited reduced functional connectivity among several key electrode sites compared to the TD group. These electrode sites included O1, O2 (occipital regions), FP1, FP2 (frontal polar regions), OZ (midline occipital region), POZ (midline parieto-occipital region), and PO3, PO4 (parieto-occipital regions). The observed reduction in connectivity suggests potential disruptions in the integration of information across occipital regions, which are critical for visual processing, and frontal polar regions, which are associated with high-level cognitive control. Furthermore, the reduced connectivity in posterior electrode sites, such as O1, O2, OZ, POZ, PO3, and PO4, indicates impairments in the visual and parieto-occipital networks, while the decreased connections in frontal polar sites, FP1 and FP2, may reflect difficulties in cognitive integration and executive functioning. These findings support the hypothesis that altered connectivity in ASD may underlie deficits in sensory processing, visuospatial integration, and higher-order cognitive functions. Overall, the results align with prior evidence of atypical functional connectivity patterns in ASD, particularly involving disrupted communication between posterior and anterior brain regions. This disruption may help explain the observed impairments in sensory integration, visuospatial processing, and cognitive regulation in individuals with ASD.

### Result of microstate

**Figure 3:**
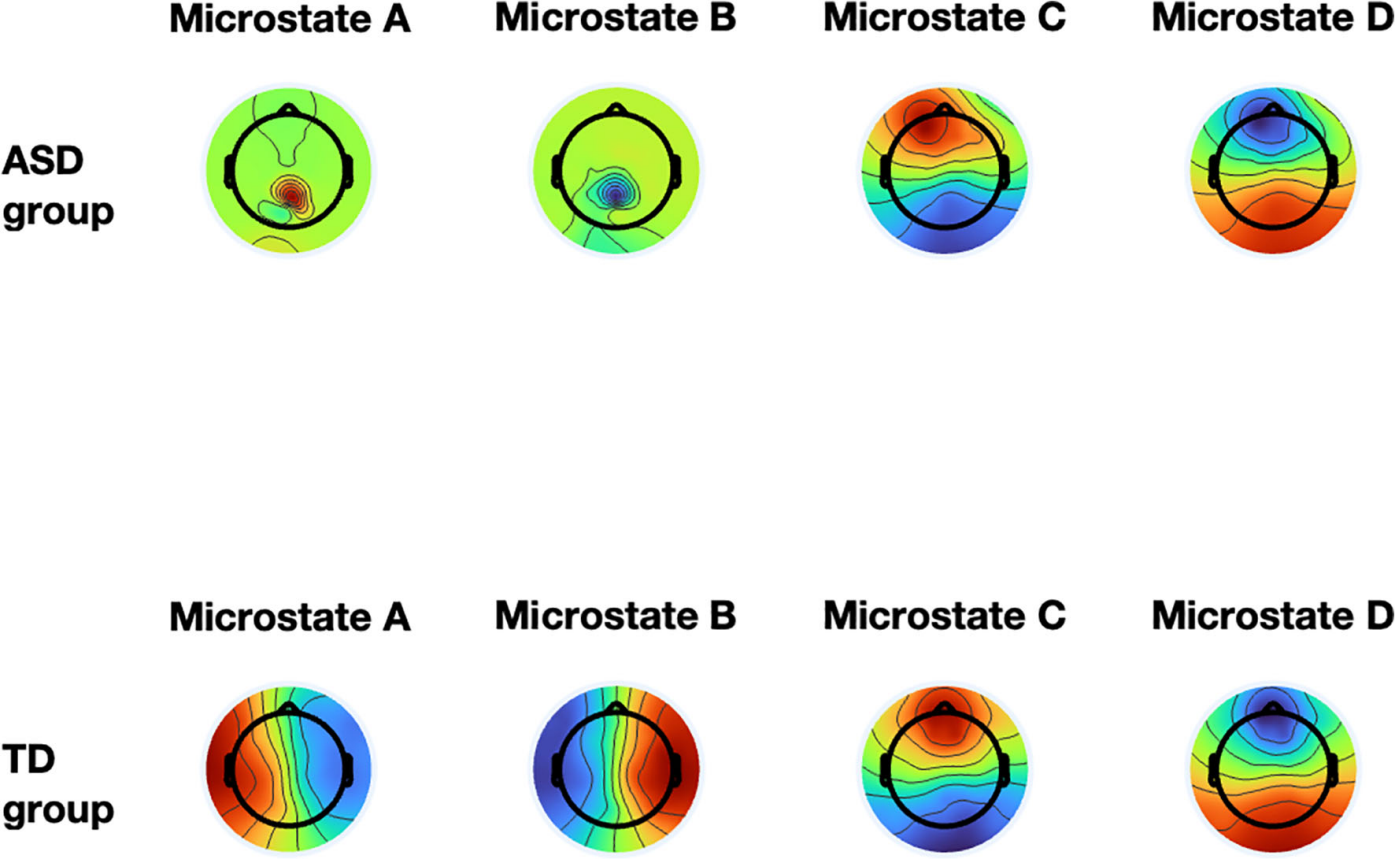
The group-level template maps of the four microstate classes. The top shows the ASD group, and the bottom shows the TD group, with the four microstate classes.

The results from all of the ANOVAs are detailed in the **Table 1**. **Figure 3** displays the differences in the template map between the ASD and TD groups. For microstate A, the TD group exhibited a significantly higher occurrence (M = 2.56 ± 0.07) compared to the ASD group (M = 1.86 ± 0.24, F(1,20) = 19.669, p < 0.001, η² = 0.376). Additionally, the ASD group demonstrated a longer duration (M = 110 ± 20.09) than the TD group (M = 96 ± 10.41, F(1,20) = 4.619, p = 0.041, η² = 0.245). The coverage of microstate A was also significantly greater in the ASD group (M = 0.17 ± 0.02) compared to the TD group (M = 0.02 ± 0.02, F(1,20) = 18.694, p < 0.001, η² = 0.571). Steady-state distribution for microstate A was substantially higher in the ASD group (M = 0.21 ± 0.02) than the TD group (M = 0.03 ± 0.02, F(1,20) = 339.547, p < 0.001, η² = 0.952).

**Table 1:** The results of the variance analysis between different class microstates are presented in **Table 1**.

class	TD group	ASD group	F_(1,20)_	P	Partial η^2^
A					
occurrence	2.56 ± 0.07	1.86 ± 0.24	19.669	0.00**	0.376
duration	96 ± 10.41	110 ± 20.09	4.619	0.041*	0.245
coverage	0.02 ± 0.01	0.17 ± 0.003	18.694	0.00**	0.571
Steady-state distribution	0.03 ± 0.02	0.21 ± 0.02	339.547	0.00**	0.952
B					
occurrence	3.38 ± 0.46	3.50 ± 0.32	0.504	0.487	0.029
duration	116.76 ± 37.28	79.82 ± 9.08	9.264	0.007**	0.353
coverage	0.39 ± 0.13	0.30 ± 0.07	4.572	0.047*	0.212
Steady-state distribution	0.40 ± 0.03	0.29 ± 0.02	83.184	0.00**	0.830
C					
occurrence	3.27 ± 0.65	3.46 ± 0.38	0.619	0.442	0.035
duration	144.63 ± 47.25	98.82 ± 22.87	7.487	0.014*	0.306
coverage	0.48 ± 0.18	0.35 ± 0.10	4.573	0.047*	0.212
Steady-state distribution	0.39 ± 0.07	0.21 ± 0.03	53.724	0.00**	0.760
D					
occurrence	1.58 ± 0.63	2.61 ± 0.56	14.306	0.001**	0.457
duration	65.79 ± 16.09	68.83 ± 3.82	0.336	0.569	0.019
coverage	0.11 ± 0.07	0.18 ± 0.05	6.655	0.019*	0.281
Steady-state distribution	0.19 ± 0.07	0.28 ± 0.03	15.375	0.001**	0.475

*p<0.05, **p<0.01.

For microstate B, the ASD group showed a significantly shorter duration (M = 79.82 ± 9.08) compared to the TD group (M = 116.76 ± 37.28, F(1,20) = 9.264, p = 0.007, η² = 0.353). Coverage of microstate B was lower in the ASD group (M = 0.30 ± 0.07) than in the TD group (M = 0.39 ± 0.13, F(1,20) = 4.572, p = 0.047, η² = 0.212). The steady-state distribution of microstate B was significantly reduced in the ASD group (M = 0.29 ± 0.02) compared to the TD group (M = 0.40 ± 0.03, F(1,20) = 83.184, p < 0.001, η² = 0.830).

For microstate C, the ASD group had a significantly shorter duration (M = 98.82 ± 22.87) compared to the TD group (M = 144.63 ± 47.25, F(1,20) = 7.487, p = 0.014, η² = 0.306). Coverage of microstate C was also lower in the ASD group (M = 0.35 ± 0.10) than in the TD group (M = 0.48 ± 0.18, F(1,20) = 4.573, p = 0.047, η² = 0.212). Furthermore, the steady-state distribution of microstate C was significantly reduced in the ASD group (M = 0.21 ± 0.03) compared to the TD group (M = 0.39 ± 0.07, F(1,20) = 53.724, p < 0.001, η² = 0.760).

For microstate D, the ASD group displayed a significantly higher occurrence (M = 2.61 ± 0.56) compared to the TD group (M = 1.58 ± 0.63, F(1,20) = 14.306, p = 0.001, η² = 0.457). Coverage of microstate D was also greater in the ASD group (M = 0.18 ± 0.05) than in the TD group (M = 0.11 ± 0.07, F(1,20) = 6.655, p = 0.019, η² = 0.281). Lastly, the steady-state distribution of microstate D was significantly increased in the ASD group (M = 0.28 ± 0.03) compared to the TD group (M = 0.19 ± 0.07, F(1,20) = 15.375, p = 0.001, η² = 0.475).

### Explained variance

**Figure 4:**
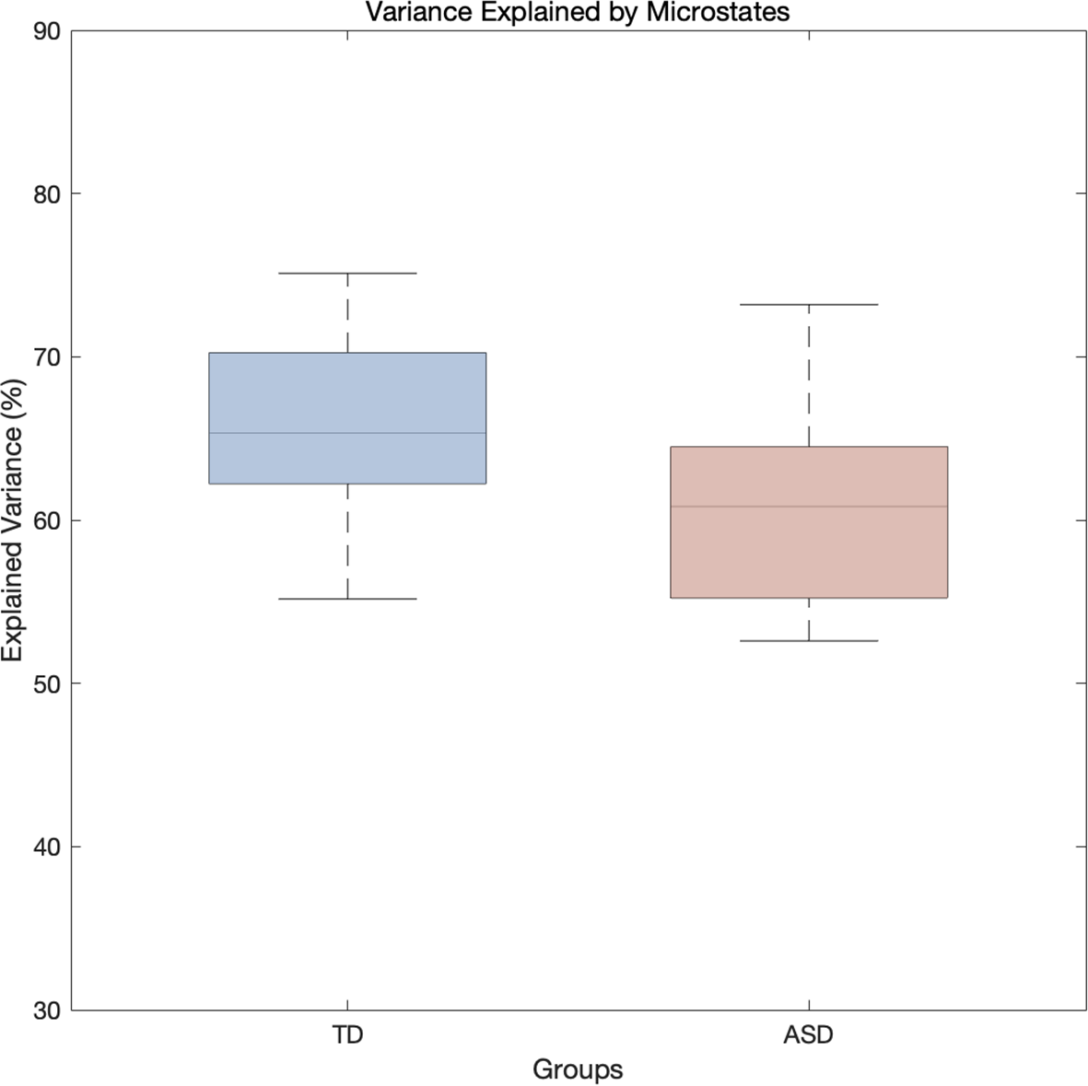
Boxplot of the variance in EEG data explained by microstates for the TD and ASD groups.

There was no significant difference in the variance of EEG data explained by microstates between the TD and ASD groups (63.9% ± 10.1% vs. 61.7% ± 7.5%; F_(1,20)_ = 0.315, *p* = 0.582, η² = 0.018). The explained variance of the data ranged from 50% to 85% as shown in **Figure 4**.

## Discussion

The results of this experiment revealed significant differences in functional connectivity and EEG microstate dynamics between the ASD group and the TD group. Specifically, the ASD group exhibited lower theta-band functional connectivity at several key electrode sites, such as the occipital, frontal polar, and parieto-occipital regions, particularly in the occipital and frontal polar regions. This may reflect impairments in sensory processing, visual processing, and higher-order cognitive control functions. Children with ASD showed significant differences in EEG microstate dynamics compared to TD controls. Specifically, the ASD group had significantly higher occurrence, coverage, and steady-state distribution in microstate A, indicating more frequent and widespread brain activity in this state. This heightened activity may be linked to overactive or distinct regulatory mechanisms in information processing. In contrast, the ASD group exhibited significantly shorter duration, lower coverage, and reduced steady-state distribution in microstate B, suggesting more transient and focused brain activity, which could reflect faster information processing but with limitations or instability. For microstate C, the ASD group showed reduced duration, coverage, and steady-state distribution, indicating more concentrated and brief brain activity, potentially reflecting constraints in sensory or cognitive processing, particularly in regions related to visual or spatial cognition. Finally, the ASD group showed significantly higher occurrence, coverage, and steady-state distribution in microstate D, suggesting more widespread and stable brain activity in this state, possibly linked to overactivity in certain cognitive functions or brain networks, particularly those involved in executive function, emotional regulation, or self-monitoring. Additionally, there was no significant difference in the explained variance between the two groups, indicating that the microstate models of both the TD and ASD groups had similar capacity in explaining the EEG data.

In this study, we investigated theta-band FC in individuals with ASD compared to typically developing TD individuals. Our analysis revealed significant differences between the two groups, particularly in regions involved in sensory processing and higher-order cognitive functions. Specifically, the ASD group showed reduced global functional connectivity, particularly in occipital and frontal polar regions, which are essential for visual processing and cognitive control. This finding is consistent with previous research that has identified disrupted communication between posterior and anterior brain regions in individuals with ASD (36, 37). When compared with prior studies, our findings align with the broader literature on ASD connectivity abnormalities. For example, one study found that ASD is characterized by long-range underconnectivity, particularly in lower-frequency bands such as theta (1, 38), which may contribute to deficits in sensory integration and executive functions. Similarly, our results show reduced connectivity in regions like the occipital and frontal polar areas, which are involved in visual processing and cognitive control, suggesting a specific disruption in long-range communication. This supports the idea that ASD involves deficits in both global connectivity and the integration of information across brain regions (39). Moreover, the observation of disrupted connectivity between posterior and anterior brain regions in our study is also reflected in findings from studies on resting-state networks. For instance, ASD has been associated with decreased functional connectivity within and between key brain networks, such as the DMN and sensorimotor network (SMN) (40). The reduced connectivity in our study in regions such as the occipital lobe and frontal polar cortex further supports this idea, indicating that ASD may be characterized by an imbalance in sensory and cognitive processing networks. Additionally, our findings are consistent with the general trend observed in other studies, where theta-band abnormalities are often linked to disruptions in executive functions and social cognition (21). The reduced theta-band connectivity in our study mirrors these findings, suggesting that these deficits in connectivity may underlie some of the cognitive and social difficulties observed in ASD, particularly in tasks requiring sensory integration and cognitive control (41). Taken together, these results add to the growing body of evidence that theta-band connectivity abnormalities play a critical role in the neurobiological underpinnings of ASD.

ASD group exhibited abnormal microstate patterns. For instance, the ASD group had a significantly higher occurrence and coverage of microstate A, but a shorter duration and reduced coverage of microstates B and C, suggesting differences in brain dynamics and information processing. Microstate A is linked to speech processing areas in the brain, such as the superior lobe and middle temporal lobe (42). In our task, the ASD group showed increased occurrence, coverage, and steady-state distribution in microstate A, which may reflect heightened or altered cognitive processing during abstract thinking. Since the task involves path selection and planning, it’s possible that individuals with ASD rely more on internal verbal thought processes, leading to more frequent brain activity in regions associated with microstate A. This suggests that the ASD group may approach abstract tasks differently, using language-based strategies to process information. The increased occurrence and steady-state distribution of microstate D in the ASD group further highlight the complex alterations in brain activity. These findings support the hypothesis that individuals with ASD exhibit atypical brain network organization, which may contribute to deficits in sensory integration, cognitive regulation, and visuospatial processing. This study revealed significant differences between the TD and ASD groups in the duration and coverage of microstates B and C, though there was no difference in their occurrence rates. These findings suggest group-specific differences in information processing. Microstate B has been primarily linked to visual processing, with its activation typically associated with enhanced activity in the visual cortex (6). This indicates that microstate B plays a key role in the reception, processing, and interpretation of visual information. In contrast, microstate C is more closely associated with self-referential processes, including self-directed cognition and autonomic processing (43). Microstate C is therefore thought to underpin higher-order cognitive functions, such as self-awareness and autonomous decision-making. While microstates B and C are individually associated with visual and self-referential processing, their interaction and combined effects on these cognitive processes warrant further exploration. These interactions may also influence more complex cognitive functions, such as attention allocation, working memory, and decision-making, thus impacting the way different groups process and understand information. The observed differences in microstate dynamics can be interpreted through the lens of “chunking dynamics,” which suggests that the brain organizes related information into larger, more meaningful units known as chunks (44). This organizational strategy reduces cognitive load and enhances processing efficiency. According to this framework, the differences in microstate duration and coverage observed in ASD children may reflect distinct chunking strategies influenced by their cognitive and experiential background. For instance, while observing the Beijing map, ASD children may have exhibited altered chunking dynamics, leading to distinct patterns of microstate activation. This aligns with cognitive schema theory, which posits that an individual’s knowledge and experiences shape their cognitive and information-processing strategies through top-down or bottom-up mechanisms (45). Differences in cognitive flexibility between the TD and ASD groups may have led to the formation of divergent cognitive schemas, as reflected in the microstate dynamics of B and C.

Recent research has increasingly focused on the application of EEG microstate analysis in understanding the neurobiological mechanisms underlying ASD. EEG microstates reflect transient patterns of brain activity and are considered potential biomarkers for neurodevelopmental disorders. Several studies have highlighted atypical microstate patterns in individuals with ASD, suggesting altered brain network connectivity (3). Specifically, disruptions in resting-state brain networks, particularly the default mode network, have been consistently reported (46, 47). The results of our EEG microstate analysis in children with ASD reveal distinct differences in the temporal and spatial dynamics of brain activity compared to TD children. These findings support the notion of atypical brain network functioning in ASD, particularly in the context of sensory processing, social cognition, and executive functioning. First, the increased duration, coverage, and steady-state distribution of microstate A in ASD children suggest a heightened persistence of brain activity in this microstate. This is consistent with previous studies, which have shown that ASD children often exhibit more sustained neural activity in certain brain regions, potentially indicating a stronger engagement with specific cognitive or sensory networks (48, 49). However, the lower occurrence frequency observed in our study could reflect a restricted capacity for dynamic switching between neural networks, which is consistent with the theory that ASD may involve impairments in the flexibility of brain network transitions (50). In contrast, microstate B and microstate C both showed shorter durations, smaller coverage, and lower steady-state distributions in the ASD group. This suggests that these microstates, typically associated with attentional processes and self-referential processing (microstate C), are less stable in ASD children. These findings are in line with literature indicating that children with ASD often struggle with attentional control, leading to less stable brain activity patterns during task processing (51, 52). The reduction in microstate C’s frequency and duration, in particular, points to disruptions in the salience network, which has been linked to difficulties in interoceptive processing in ASD (50). On the other hand, microstate D demonstrated a higher occurrence frequency, longer duration, and stronger steady-state distribution in the ASD group, suggesting that brain activity in this microstate is more stable and sustained. This aligns with earlier studies which suggest that certain brain regions may exhibit more robust activity patterns in ASD, possibly reflecting compensatory mechanisms or altered regulation in specific neural circuits (50, 53). This could be linked to enhanced stability in executive functions, although the specific neural mechanisms underlying these changes remain unclear.

In this study, we focused on the brain’s microstate dynamics and theta-band FC during a route-planning task, which adds a novel perspective to previous research on spatial navigation in ASD. While earlier studies have explored various aspects of spatial navigation in ASD, such as allocentric and egocentric learning (54) and cognitive flexibility in complex environments (55), our task integrates both active route planning and passive observation, offering a more ecological approach to studying decision-making in navigation. By combining these elements with EEG data, we aim to link specific neural patterns, including microstates and theta-band FC, to navigational abilities and decision-making processes. Previous research has highlighted the involvement of theta oscillations in higher-order cognitive processes, including memory and attention, which are key components of navigation tasks (55, 56). Our task, which involves selecting the most efficient route under time constraints, likely engages these cognitive systems, providing insight into how individuals with ASD process spatial information differently. In particular, we observed significant differences in theta FC between ASD and TD groups, especially in regions related to sensory processing and higher-order cognitive functions. These differences in theta oscillations and microstate A dynamics are consistent with findings from prior research, where altered connectivity patterns were associated with difficulties in switching between cognitive states (3). Moreover, by examining microstate dynamics, our study extends previous work by offering a more nuanced view of brain activity during the navigation process. Microstate A, which is associated with speech processing and cognitive control (47), showed heightened occurrence and coverage in the ASD group, possibly indicating more frequent or distinct processing of navigational information. This finding aligns with theories suggesting that ASD may involve heightened local processing or alternative cognitive strategies (1, 2). Our design thus offers a unique opportunity to link specific microstate dynamics and theta-band connectivity to the behavioral outcomes observed in spatial navigation tasks, providing a deeper understanding of the neural mechanisms underlying ASD’s navigation-related challenges. In conclusion, the combination of microstate analysis and theta FC in our route-planning task enhances the understanding of the neural underpinnings of spatial navigation in ASD. By integrating both behavioral and neural perspectives, this approach contributes to the growing body of research on how ASD individuals process and navigate complex environments, offering novel insights into the relationship between cognitive flexibility, attentional control, and neural connectivity. This approach also lays the groundwork for future studies to explore how these neural dynamics may relate to real-world navigation and decision-making in individuals with ASD.

From the perspective of Lehmann’s framework, microstates can be considered fundamental units of human information processing, reflecting the interaction between environmental stimuli and the individual’s prior knowledge and internal states (57). The differences in the duration and coverage of microstates B and C observed in this study could be interpreted as outcomes of such interactions. Specifically, the participants’ cognitive background and internal states may have influenced their approach to observing and processing the Beijing map, leading to group-specific patterns in microstate transitions. Lehmann’s theory underscores the importance of the interplay between environmental stimuli and individual factors in shaping information processing and microstate dynamics (6, 58). These findings suggest that the activated microstates reflect functional brain states modulated by participants’ knowledge and experience, potentially impacting their behavior and information-processing strategies. The heteroclinic channel theory provides an additional lens to interpret these results. This theory posits that a system transitions between stable states (attractors) via specific pathways, referred to as heteroclinic channels (59, 60). In the context of our study, the microstates A, B, C, and D can be viewed as distinct attractors, and the differences in their duration and coverage reflect the properties of these transitions. The lack of significant differences in occurrence rates suggests that participants may frequently switch between microstates B and C, leading to balanced counts but differing durations and coverage. This implies that the dynamics of microstate transitions, rather than their frequencies, are critical in differentiating the TD and ASD groups during visual map processing. Importantly, functional connectivity (FC) analysis complements these findings. The FC results showed that, compared to the TD group, ASD children exhibited reduced connectivity among key electrodes, including O1, O2, FP1, FP2, OZ, POZ, PO3, and PO4, during the navigation task. These electrodes are closely linked to visual and frontoparietal networks, suggesting disruptions in the coordinated activity required for effective visuospatial processing and self-referential tasks. The reduced FC in these regions aligns with the microstate findings, as impairments in microstate B (associated with visual processing) and C (linked to self-directed cognition) could reflect and contribute to diminished functional integration across these networks.

Overall, our findings contribute to the growing body of evidence that ASD is characterized by unique patterns of brain activity, especially in the temporal dynamics of EEG microstates. The results suggest that children with ASD have altered brain network connectivity, particularly in regions responsible for sensory processing and executive functions. These alterations are likely to reflect the broader neurodevelopmental and cognitive challenges observed in ASD. This study on navigation EEG microstates in ASD is particularly innovative as it builds upon this foundational research while addressing key gaps in the literature. Specifically, the exploration of microstates in the context of navigation—a higher-order cognitive process—represents an untapped area of investigation. By extending microstate research to include task-based EEG rather than just resting-state data, our work has the potential to elucidate how atypical neural activity manifests in real-world behaviors, offering novel insights into ASD. This innovation not only enhances the understanding of ASD-related neural dysregulation but also opens pathways for applications in personalized interventions. Based on recent findings regarding abnormal microstates and weakened theta functional connectivity in children with ASD, several promising interventions have emerged. Neurofeedback training, which targets specific EEG patterns such as theta waves, has shown potential in improving brain connectivity and social behavior in ASD (61). Cognitive training programs, particularly those focused on enhancing executive functions, can also help re-regulate neural dynamics (62). Additionally, virtual reality (VR) training has been shown to improve social skills and executive functions in ASD (63), potentially helping to recalibrate microstate abnormalities and enhance theta connectivity. Finally, transcranial direct current stimulation (tDCS) has demonstrated its ability to modulate brain connectivity, offering a targeted intervention for ASD-related microstate abnormalities (48). Building on the results of this study, future research could leverage these techniques to intervene in children with ASD, aiming to enhance theta connectivity and improve microstate abnormalities. These approaches hold promise for addressing the neural disruptions underlying ASD. Together, the findings from microstate and functional connectivity analyses provide converging evidence that children with ASD exhibit atypical neural dynamics during complex tasks like visuospatial navigation. The observed abnormalities in both microstate metrics and functional connectivity underscore the multifaceted nature of neural disruptions in ASD. These findings highlight the interplay between disrupted global connectivity and altered temporal dynamics, offering new insights into the neurophysiological basis of cognitive impairments in ASD. Future research that integrates these modalities may further clarify the relationship between temporal and spatial brain dynamics in ASD and their impact on cognitive processing.

## Data Availability

The raw data supporting the conclusions of this article will be made available upon reasonable request.
